# The Effects of Phthalates on the Ovary

**DOI:** 10.3389/fendo.2015.00008

**Published:** 2015-02-02

**Authors:** Patrick R. Hannon, Jodi A. Flaws

**Affiliations:** ^1^Department of Comparative Biosciences, University of Illinois at Urbana-Champaign, Urbana, IL, USA

**Keywords:** phthalates, phthalic acid, ovary, female reproductive toxicology, ovarian toxicology, folliculogenesis, steroidogenesis

## Abstract

Phthalates are commonly used as plasticizers in the manufacturing of flexible polyvinyl chloride products. Large production volumes of phthalates and their widespread use in common consumer, medical, building, and personal care products lead to ubiquitous human exposure via oral ingestion, inhalation, and dermal contact. Recently, several phthalates have been classified as reproductive toxicants and endocrine-disrupting chemicals based on their ability to interfere with normal reproductive function and hormone signaling. Therefore, exposure to phthalates represents a public health concern. Currently, the effects of phthalates on male reproduction are better understood than the effects on female reproduction. This is of concern because women are often exposed to higher levels of phthalates than men through their extensive use of personal care and cosmetic products. In the female, a primary regulator of reproductive and endocrine function is the ovary. Specifically, the ovary is responsible for folliculogenesis, the proper maturation of gametes for fertilization, and steroidogenesis, and the synthesis of necessary sex steroid hormones. Any defect in the regulation of these processes can cause complications for reproductive and non-reproductive health. For instance, phthalate-induced defects in folliculogenesis and steroidogenesis can cause infertility, premature ovarian failure, and non-reproductive disorders. Presently, there is a paucity of knowledge on the effects of phthalates on normal ovarian function; however, recent work has established the ovary as a target of phthalate toxicity. This review summarizes what is currently known about the effects of phthalates on the ovary and the mechanisms by which phthalates exert ovarian toxicity, with a particular focus on the effects on folliculogenesis and steroidogenesis. Further, this review outlines future directions, including the necessity of examining the effects of phthalates at doses that mimic human exposure.

## Phthalates

**Figure 1:**
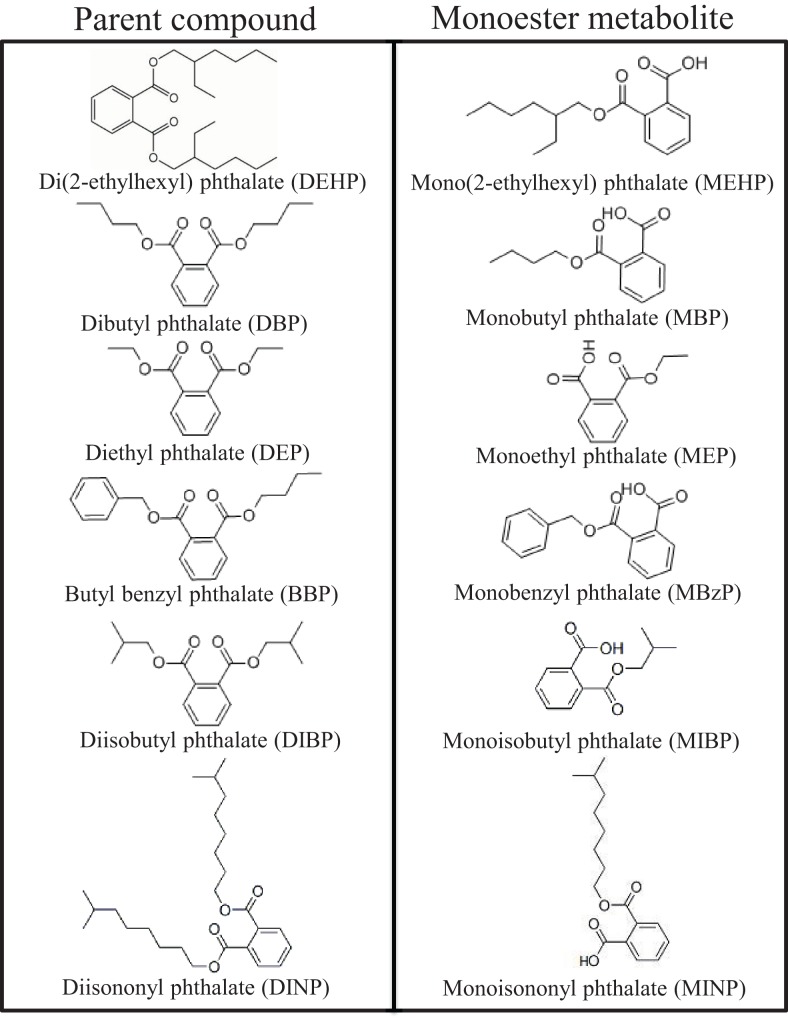
**Chemical structures of common phthalates and their monoester metabolites that are mentioned in this review**.

Phthalates are ubiquitous environmental toxicants to which humans are exposed on a daily basis (1). They are a group of synthetic chemicals composed of alkyl diesters of phthalic acid and are named based on their varying lengths of alkyl chains (Figure 1). Normally, phthalates in their pure form are colorless, odorless, oily liquids with high lipophilic properties, and low solubility in water. Phthalates are predominantly used as plasticizers in polyvinyl chloride consumer, medical, and building products to impart flexibility, as matrices and solvents in personal care products, and as excipients in medications and dietary supplements. As plasticizers, phthalates are present in commonly used items such as flooring, roofing, carpeting, shower curtains, packaging equipment, food and beverage packaging, automotive parts, and even in children’s toys. Interestingly, di(2-ethylhexyl) phthalate (DEHP) is present in common medical devices such as tubing, blood and intravenous bags, dialysis equipment, and in the manufacturing of disposable and surgical gloves (2). As matrices and solvents, phthalates are commonly found in consumer and cosmetic products ranging from hairsprays and perfumes to pesticides and wood finishes. Further, they are frequently used as adhesives, defoaming agents, and lubricants (3). As excipients, some phthalates are incorporated in the enteric coating of oral medications and in dietary supplements ranging from certain fish oils to probiotics (4, 5). Thus, there are multiple means of phthalate exposures due to their presence in a wide range of products used by humans on a daily basis.

Daily exposure to phthalates is also attributed to their widespread production. The global production and use of phthalates exceeds 18 billion pounds per year, in which the majority of phthalates are used in polyvinyl chloride products (6). The most commonly used phthalate is DEHP, which belongs to a group of phthalates known as dioctyl phthalates. Domestic production of dioctyl phthalates exceeds 300 million pounds annually (7). Dibutyl phthalate (DBP) and diethyl phthalate (DEP) are also produced in high volumes and are among the most commonly used phthalates in consumer products. Production and importation of DBP was estimated to be between 10 and 50 million pounds in the United States in 2006 (8). Further, production of DEP reached 50 million pounds in the United States in 2005 (9).

Phthalates are non-covalently bound to plastics, meaning they frequently leach from these items into environmental sources such as in the atmosphere, soil and sediments, and natural water bodies (10–12). Phthalate contamination in the air can range from 1 to 50 ng/m^3^, but DEHP is often found at higher levels (up to 3640 ng/m^3^) (10). Once in the air, phthalates typically bind to dust particles and are carried back to ground level (7). Phthalates are also detectible in sediments (0.01–115 mg/kg), agricultural soil (0.02–264 mg/kg), and urban soil (0.01–30.1 mg/kg) (10). Urban sewer wastewater is another source of phthalate accumulation in the environment. Median phthalate levels are 3.46 μg/l in industrial wastewater, 61.3 μg/l in residential wastewater, and 66.0 μg/l in man-made wastewater (10). Further, phthalates are found in surface water, including freshwater, saltwater, and industrial water, at levels ranging from 0.29 to 1.24 μg/l (10, 13–15). Based on their presence in natural water bodies, fish and other aquatic animals are also exposed to phthalates. Although few studies have measured the levels of phthalates in fish populations, levels of DEHP and DBP in freshwater fish are 1.8 μg/kg each (16). Further, levels of phthalates in saltwater biota range from 0.0022 to 28.7 μg/g, and interestingly, phthalates do not appear to accumulate in trophic positions (17). Although few studies have investigated the lifespan of phthalates in the environment, phthalates are considered fairly stable in the environment and can persist for quite a long time (7, 18–20). Phthalates in the air and soil dissolve very slowly, whereas phthalates in surface water dissolve quicker in water with a half-life of 2–3 weeks (7, 18–20).

The widespread production of phthalates, their use in commonly used products, and their presence in the environment leads to daily human exposure via oral ingestion, inhalation, and dermal contact. The most common routes of exposure are via oral ingestion from food packaging and use of cosmetic products, but high levels of phthalates are also present in household dust (21, 22). Based on large production volumes, widespread use, and environmental contamination, biomonitoring data suggest that 75–100% of the population is exposed to phthalates on a daily basis (23–25). Thus, exposure to phthalates is ubiquitous in human populations.

Once consumed, phthalates are rapidly metabolized in the gut, liver, and blood by esterases and lipases. Initially, the phthalate diester is cleaved to its respective hydrolytic monoester where only one alkyl chain remains on the phthalic acid backbone, and interestingly, it is often the monoester metabolites that induce toxicity. Depending on the size of the remaining monoester metabolite, the alkyl chain can undergo further oxidative metabolism and ultimately glucuronidation depending on the species (21, 26). These hydrolytic monoester and oxidative monoester metabolites, in addition to the parent phthalates, are used as biomarkers to estimate daily human exposure levels (26). Careful attention to the metabolite used for biomonitoring is essential for accurate estimations of daily exposure levels. For example, it is more accurate to measure oxidative monoester metabolites from high molecular weight phthalates, such as DEHP, than it is to measure the hydrolytic monoester metabolite (26). In one study, the concentrations of the oxidative monoester metabolites of DEHP, mono(2-ethyl-5-oxohexyl) phthalate and mono(2-ethyl-5-hydroxyhexyl) phthalate, were found to be four-fold higher than the hydrolytic monoester metabolite, mono(2-ethylhexyl) phthalate (MEHP) (27). Thus, some metabolites are more sensitive biomarkers than others.

As mentioned, the vast majority of the population is exposed to phthalates on a daily basis, but the level of exposure to each phthalate differs. It is estimated that the average total daily individual ambient exposure to DEHP ranges from 0.21 to 2.1 mg/day for the general population (28–32). Thus, the estimated range of daily human exposure to DEHP is 3–30 μg/kg/day based on urinary metabolite concentrations; however, measurements of DEHP in household dust can reach up to 700 mg/kg, potentially increasing exposure levels in certain individuals (22, 29, 33). Koch and Calafat compiled data from the United States and German populations where urinary metabolites were used to estimate daily exposure levels for other commonly used phthalates, such as DEP, butyl benzyl phthalate (BBP), DBP, and diisobutyl phthalate (DIBP). The estimated range of daily human exposure to DEP is 2.32–12 μg/kg/day, BBP is 0.26–0.88 μg/kg/day, DBP is 0.84–5.22 μg/kg/day, and DIBP is 0.12–1.4 μg/kg/day (26). Based on these exposure levels, phthalates have been identified as top contaminants present in human tissues. As stated above, measureable levels of phthalates are found in human urine samples tested and in 95% of human blood samples tested (1, 22, 23, 25, 34). In particular to reproduction and development, DEHP and its metabolites are present in 90–100% of amniotic fluid samples from second trimester fetuses, cord blood samples from newborns, breast milk from nursing mothers, and even in human ovarian follicular fluid, indicating their ability to reach the ovary (1, 23, 34, 35).

Interestingly, certain individuals are exposed to much higher levels of phthalates than the general population. Not surprisingly, the levels are much higher in humans occupationally exposed to phthalates. For example, it was estimated that in the 1980s, over 340,000 and 239,149 workers were exposed to DEHP and DEP, respectively (36). Further, the exposure level of DEHP to these workers was between 143 and 286 μg/kg/day (37).

The highest exposures to phthalates often result from medical therapies. Both DEP and DEHP are incorporated in medical equipment, and DEP and DBP can be found in the enteric coating of oral medications. Based on its use in medical equipment, levels of DEHP can reach 8.5 mg/kg/day following blood transfusions, 0.36 mg/kg/day following hemodialysis, and 14 mg/kg/day following extracorporeal membrane oxygenation procedures in neonates (38). Additionally, infants in intensive neonatal care units had levels of DEHP metabolites that were 14 times higher than infants in a low-intensive unit (39). Based on their use in oral medications, urinary levels of monoethyl phthalate (MEP), the monoester metabolite of DEP, and monobutyl phthalate (MBP), the monoester metabolite of DBP, in women of childbearing age were over 12 and 200 times higher, respectively, than in a reference population (40). In another study, urinary measurements of MBP were 50 times higher in subjects that reported using oral medications containing DBP than in controls (5).

Important for the topic of this review, women have a phthalate exposure profile that is different than that in men. In fact, females at all ages have increased urinary phthalate metabolite levels when compared to men at that same age (23). Compared to males, females have higher levels of MEP, MBP, monobenzyl phthalate (MBzP), and MEHP (23). Interestingly, women of reproductive age have the highest exposure levels of MBP than any other age/sex group (41). These findings are likely attributed to the widespread use of phthalates, in particular MBP, in common cosmetic and personal care products that females use on a daily basis, including perfume, lotion, nail polish, and hairspray.

Exposure to phthalates is a public health concern because several have been identified as reproductive and developmental toxicants and endocrine-disrupting chemicals (EDCs). In females, chronic occupational exposure to high levels of phthalates has been associated with decreased rates of pregnancy and high rates of miscarriage (1, 42). Further, high urinary phthalate levels are associated with pregnancy complications such as anemia, toxemia, and preeclampsia in women (43). In laboratory animals, phthalates reduce implantations, increase resorptions, decrease fetal weights of offspring, and decrease incidence of pregnancy (44, 45). The mechanisms by which phthalates disrupt these endocrine and reproductive events remain unknown. Interestingly, the ovary is a critical regulator of these processes, and the effects of phthalates on ovarian function remain poorly understood. The next sections will provide background on the importance of normal ovarian function for reproductive and non-reproductive health and how EDCs, like phthalates, can disrupt ovarian function.

## The Ovary

The ovary is the female gonad responsible for reproduction and is a primary component of the female endocrine system. This heterogeneous organ is comprised of a surface epithelium surrounding the ovary, an outer cortex region containing ovarian follicles, corpora lutea, and stroma, and an inner medulla region containing a vast network of blood vessels, lymphatic vessels, and nerves. The main functions of the ovary include maturation and ovulation of the female gamete (oocyte) for fertilization and secretion of sex steroid hormones necessary for reproductive and non-reproductive health.

## Folliculogenesis

**Figure 2:**
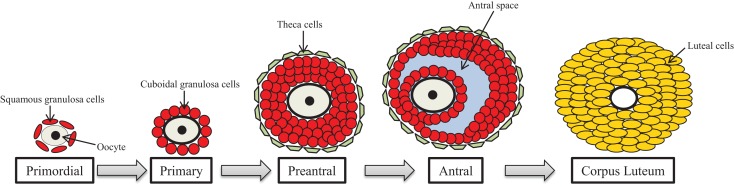
**Ovarian folliculogenesis**. The female is born with a finite number of primordial follicles that can mature through the primary, preantral, and antral stages of development. The follicle contains the gamete (oocyte) surrounded by granulosa cells (shown in red) and theca cells (shown in green), which are somatic cells. Following ovulation, the antral follicle differentiates into the corpus luteum, and the granulosa and theca cells become large and small luteal cells, respectively.

One of the primary functions of the ovary is the development and maturation of follicles to allow for ovulation of the oocyte for subsequent fertilization. The ovarian follicle is the functional unit of the ovary that consists of the oocyte surrounded by two somatic cell types termed the granulosa cells and the theca cells. Within the ovarian unit, follicles undergo several irreversible developmental transitions, and this process of follicular development is known as ovarian folliculogenesis (Figure 2).

In mammals, the female is born with a finite number of follicles; thus, the follicular reserve is set at birth and represents a female’s reproductive potential and reproductive lifespan (46). These follicles are first formed during the later stages of fetal life in the human and during the early post-natal life in the rodent. The process of follicle formation is known as germ cell nest breakdown. During embryonic development, primordial germ cells, which will give rise to oocytes, migrate from the yolk sac to the genital ridge where the undifferentiated gonad resides (47). These germ cells, now termed oogonia, massively proliferate via mitosis and develop in clusters or nests in which squamous pre-granulosa cells surround the oogonia (48). Once established in the germ cell nests, mitosis of oogonia is ceased and meiosis begins. It is here that the oogonia become oocytes, and the oocytes progress through meiosis until they are arrested in the diplotene stage of meiotic prophase I (49).

Ovarian follicle assembly then occurs around the sixth to ninth month of gestation in the human and around post-natal day 3 in the rodent in which the most immature follicle type, the primordial follicle, is formed (50, 51). For primordial follicle assembly to occur, the germ cell nests must undergo programed cell death of oocytes, primarily through regulation of the B-cell lymphoma/leukemia-2 (BCL-2) family members (52–56) and the actions of steroid hormone and intraovarian growth and transcription factors (57, 58). The interaction of these molecular events leads to oocyte association with a single layer of flattened, squamous pre-granulosa cells, thus, the formation of the primordial follicle.

Once the primordial follicle population is established, the follicle is destined to three fates: to remain quiescent for varying lengths of time to constitute the ovarian reserve, to directly undergo atresia, which is follicular programed cell death via apoptosis, or to activate into the growing population of follicles to become primary follicles, a process termed primordial follicle recruitment. Primordial follicle recruitment is a tightly regulated process controlled by multidirectional communication between the oocyte, granulosa cells, and surrounding somatic cells that will give rise to the theca cells. This process is gonadotropin-independent and relies on paracrine and autocrine regulation by multiple intrinsic ovarian growth factors that work through several different signaling pathways (46, 58–61). Primordial follicle quiescence is maintained by factors that suppress follicle activation, whereas primordial follicle recruitment is initiated by factors that activate development. These stimulatory and inhibitory factors exist in a balance to maintain primordial follicle survival so that downregulation of inhibitory factors and/or overactivation of stimulatory factors favor an environment conducive for primordial follicle recruitment (62).

Once activated, primary follicles contain a larger oocyte that has initiated growth surrounded by a single layer of cuboidal granulosa cells. Primary follicles then develop into preantral follicles, also termed secondary and tertiary follicles that contain the oocyte surrounded by at least two layers of cuboidal granulosa cells and two outer theca cell layers. Follicles at this stage, because of the presence of both granulosa and theca cells, are gonadotropin-responsive and begin synthesizing sex steroid hormones.

Preantral follicles then develop further into antral follicles, which are the most mature follicle type in the ovary. Antral follicles contain the oocyte surrounded by several layers of cuboidal granulosa cells with a fluid filled space, termed the antral space, and two outer theca cell layers.

Each fertile menstrual/estrous cycle requires the presence of a pre-existing antral follicle population that responds to cyclic gonadotropins, and this process is termed cyclic recruitment (60). Therefore, folliculogenesis must remain dynamic to allow for the continual generation of antral follicles to undergo cyclic recruitment for potential ovulation. As antral follicles continue to mature, they produce estradiol and their receptivity to the gonadotropins, follicle-stimulating hormone (FSH) and luteinizing hormone (LH), increases. The increase in estradiol initiates the LH surge causing one or multiple follicles to ovulate depending on the species. Once the oocyte is released, the remaining granulosa and theca cells differentiate into large and small luteal cells respectively, and the remaining structure is termed the corpus luteum.

Not all follicles are destined to develop and ovulate, and in fact, approximately 99% of follicles undergo atresia. At birth, the human ovary contains approximately two million follicles, but by puberty, the number of follicles declines to roughly 400,000 due to atretic demise. Further, of the available follicles at puberty, only about 400 of them will ovulate throughout the reproductive lifespan, whereas the others undergo atresia (50). Atresia is a coordinated process of follicle degeneration via hormonally controlled apoptosis (63). Although atresia can occur at all stages of follicle development, early antral follicles are most susceptible to death in which apoptosis can occur in both somatic and germ cells. The regulation of follicular atresia involves a balance of pro- and anti-apoptotic factors. Specifically, gonadotropins, estrogens, insulin-like growth factor-I, and interleukin-1β are anti-apoptotic and help prevent follicles from undergoing atresia (64–70). Conversely, tumor necrosis factor-α, Fas–Fas ligand, and androgens promote apoptosis and ultimately atresia (71–74). The interplay of these pro- and anti-apoptotic factors primarily converges on the BCL-2 signaling pathway, with its own pro- and anti-apoptotic proteins, to regulate atresia (53–56).

## Ovarian Steroidogenesis

**Figure 3:**
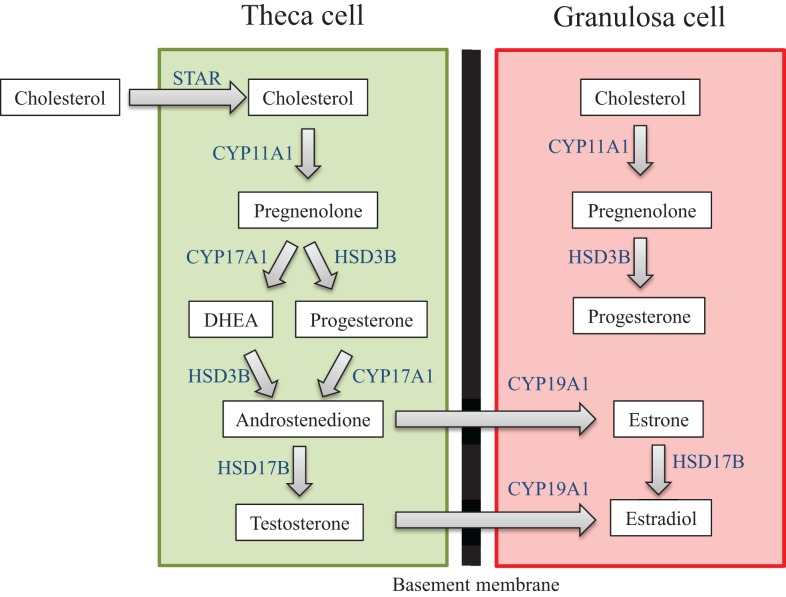
**Ovarian steroidogenesis**. Steroidogenesis is primarily conducted by the mature antral follicle and the corpus luteum following ovulation. This process requires both the theca cells and granulosa cells, and involves the enzymatic conversion of cholesterol to 17β-estradiol and other necessary sex steroid hormones. The hormones produced by the ovary are listed in the white text boxes while the steroidogenic enzymes are listed in blue adjacent to the arrows between hormones.

Another primary function of the ovary is to produce sex steroid hormones, a process termed ovarian steroidogenesis. Steroidogenesis is primarily conducted by the mature antral follicle and the corpus luteum following ovulation. The process of steroidogenesis involves the enzymatic conversion of cholesterol to 17β-estradiol and other necessary sex steroid hormones to regulate reproductive and non-reproductive health (Figure 3). Prior to the peri-ovulatory period, the antral follicle increases the synthesis of estradiol to promote the ovulatory surge of LH. As ovulation approaches, the peri-ovulatory follicle increases production of progesterone to promote ovulation and formation of the corpus luteum. Once the follicle has transitioned to the highly vascularized corpus luteum, vast amounts of progesterone as well as estradiol are produced.

The steroid hormones produced by the ovary act on numerous target tissues associated with reproductive and non-reproductive function. For reproductive function, steroid hormones act on the ovary itself as well as the brain, pituitary, oviduct, uterus, cervix, vagina, and mammary gland. The actions of these steroid hormones include maintenance of the reproductive tract; establishment of a hormonal milieu for ovulation, fertilization, implantation, and pregnancy; and control of menstrual/estrous cyclicity by utilizing feedback loops in the brain and pituitary. These steroid hormones also act in non-reproductive tissues such as the brain, cardiovascular system, adipose tissue, skin, bone, and liver. Therefore, proper steroidogenesis is required for fertility as well as for maintenance of cardiovascular, brain, and skeletal health (75–91).

The generation of sex steroid hormones involves several enzymatic reactions in both the theca and granulosa cells. Specifically, cholesterol can either be transported into the theca cell cytoplasm via lipoprotein receptors or it can be synthesized *de novo*. Cholesterol is then internalized into the mitochondria via the steroidogenic acute regulatory protein (STAR) (92–94). Cholesterol is then converted to pregnenolone in the mitochondria via cytochrome-P450 cholesterol side-chain cleavage (CYP11A1) (95, 96). Pregnenolone then diffuses out of the mitochondria and is transported to the smooth endoplasmic reticulum where it is converted to progesterone or dehydroepiandrosterone (DHEA) via 3β-hydroxysteroid dehydrogenase (HSD3B) or 17α-hydorxylase-17,20-desmolase (CYP17A1), respectively (97). Progesterone and DHEA are then converted to the androgen androstenedione again via CYP17A1 or HSD3B, respectively (96). Androstenedione can then be converted to either testosterone, another androgen, or estrone, a weak estrogen, via 17β-hydroxysteroid dehydrogenase (HSD17B) or aromatase (CYP19A1), respectively (96). Testosterone and estrone are then converted to the most potent estrogen, estradiol, via CYP19A1 or HSD17B, respectively (96, 97). Estradiol can be inactivated and metabolized in the ovary to 2-hydroxyestradiol via CYP1A1/2 and CYP3A4 or it can be broken down to 4-hydroxyestradiol via CYP1B1 (98, 99).

Interestingly, estradiol cannot be synthesized without the strict coordination of both theca cells and granulosa cells and the addition of pituitary-derived FSH and LH. This is why ovarian steroidogenesis is known as the two-cell, two-gonadotropin theory (100, 101). Theca cells in the early antral follicle only contain LH receptors (LHRs), and upon receptor binding, LH stimulates the transcription of theca-derived genes that encode the enzymes required for the conversion of cholesterol to the androgens (100, 101). Once converted, androgens can diffuse from the theca cells through the basement membrane, which separates theca cells from granulosa cells, and into the granulosa cells. In contrast to theca cells, granulosa cells of the early antral follicle contain only FSH receptors, and in response to FSH binding, the transcription of granulosa-derived genes that encode the enzymes necessary for the conversion of androgens to estrogens is stimulated (100, 101). This distinct coordination is required because theca cells lack the CYP19A1 enzyme (which converts androgens to estrogens), and granulosa cells lack the CYP17A1 enzyme (which converts pregnenolone and progesterone to androgens). Luteal cells in the corpus luteum also utilize the two-cell approach to produce progesterone and estradiol (102, 103). As is the case in the antral follicle, the small luteal cells that are derived from theca cells synthesize androgens from cholesterol, while the large luteal cells that are derived from granulosa cells convert androgens to estrogens.

## Ovarian Toxicity of Endocrine-Disrupting Chemicals

Because of its multifaceted roles, it is important to understand how ubiquitous EDCs, like phthalates, affect normal ovarian function, as defects in ovarian function have implications for health other than fertility. Normal ovarian function is essential for reproductive, cardiovascular, mood, brain, and skeletal health (75–91). Due to their widespread production, extensive use, and ubiquitous presence in the environment, phthalates have the potential to target the ovary at all stages of development and in adulthood. These toxic effects can lead to premature ovarian failure, anovulation, infertility, and decreased steroidogenesis (104–107). Thus, exposure to phthalates can disrupt normal ovarian function by several different mechanisms, leading to reproductive and non-reproductive abnormalities.

One way that EDCs can exert ovarian toxicity is through targeting follicles at different stages of folliculogenesis (104–107). Specifically, chemicals can target the primordial, primary, preantral, or antral populations of follicles, or they can target corpora lutea. Once a particular population is targeted, the chemicals can induce atresia and deplete the follicles within that stage, they can arrest follicles within that stage, or they can promote accelerated development from that stage (104–107). Each of these potential outcomes can have detrimental effects on fertility and/or non-reproductive health. Specifically, EDCs that deplete or accelerate the development of primordial follicles will cause permanent infertility caused by premature ovarian failure, or early onset of menopause (104–107). This is because the primordial follicle pool is established at birth and is non-renewable (46). Premature menopause is of concern because it is associated with increased risks of cardiovascular disease, osteoporosis, and premature death (75–78, 108–111). EDCs can also target the later stages of folliculogenesis such as the antral follicle (104, 105). Chemicals can cause atresia of antral follicles or inhibit the growth of antral follicles, leading to estrogen deficiency and anovulatory cycles and ultimately infertility (104, 105). Similarly, EDCs that affect the process of luteinization, the developmental transition of a follicle to a corpus luteum, or the lifespan of the corpus luteum can affect progesterone production, implantation, and pregnancy, leading to infertility (104, 105).

Chemicals can also directly interfere with ovarian steroidogenesis and this can cause reproductive and non-reproductive complications. Steroidogenesis can be affected either by depletion of the antral follicles and/or corpora lutea, or it can be affected by disrupting the functionality of the steroidogenic units. Specifically, the loss of antral follicles or corpora lutea from the ovary will result in a decrease in the available structures that are capable of producing steroids (104, 105). Further, EDCs can disrupt the functionality of antral follicles by decreasing ovarian mRNA, protein, and/or activity of the enzymes responsible for generating estradiol and its precursor sex steroid hormones (104, 105). Additionally, EDCs can increase mRNA, protein, and/or activity of the enzymes responsible for metabolizing estradiol; thus, rendering it inactive (104, 105). The steroidogenic enzymes in the corpora lutea can also be affected in a similar manner, resulting in inadequate levels of necessary progesterone and estradiol to support a pregnancy (104, 105). This disruption of hormone production can also alter normal menstrual/estrous cyclicity. A lack of ovarian-derived steroid hormones will disrupt the hypothalamus–pituitary–ovarian axis, leading to infertile anovulatory or oligoovulatory cycles by inhibiting the LH surge and/or altering FSH levels that are responsible for recruiting a cohort of antral follicles for ovulation (46, 101, 104, 105). Defects in ovarian steroidogenesis are linked to infertility and an increased risk of heart disease, osteoporosis, mood disorders, and premature death (75–78, 108–111).

Removing or minimizing toxicant exposure may alleviate the ovotoxic effects, depending on the duration of exposure and population of follicles targeted by the chemical (106, 107). For instance, if an EDC only targets the antral follicle causing ovarian toxicity, removal of the chemical has the potential to restore ovarian function (106, 107). This is because the process of folliculogenesis from the primordial stage to the antral stage was unaffected. Once the EDC is removed, primordial follicles will develop to the antral stage as they had done previously, but now the detrimental effect will be alleviated (106, 107). Reversal of toxic effects is nearly impossible when the primordial follicle pool is targeted for an extended period of time. Because the follicular reserve is non-renewable, chronic exposure to an EDC that causes death of primordial follicles or accelerates primordial follicle recruitment will lead to permanent ovarian damage (106, 107). Although removal of toxicant exposure may be beneficial in restoring ovarian function, exposure to many EDCs including phthalates cannot be completely removed. This adds to the public health concern of the use of phthalates, as even minimizing exposure can be a difficult task due to their ubiquitous use in common consumer products and presence in the environment.

Understanding the impact on ovarian function from exposure to phthalates is of great importance, particularly because the general population is constantly exposed to phthalates (23–25). This importance is compounded in certain populations that are exposed to high levels of phthalates on a daily basis. These populations include patients undergoing medical care with phthalate containing medical devices and medications, women with careers in an industrialized environment, and women located near phthalate manufacturing and disposal sites (5, 36–41). Often, women in today’s society postpone childbirth to prioritize career development during prime reproductive years. This leads to a longer period of exposure to phthalates, potentially leading to detrimental effects on fertility, especially when the female is aging. Because of the prevalent use and ubiquitous exposure to phthalates and the importance of the ovary for female reproductive and non-reproductive health, the goal of this review is to summarize what is currently known about the effects of phthalates on the ovary and the mechanisms by which phthalates exert ovarian toxicity, with a particular focus on the effects of phthalates on folliculogenesis and steroidogenesis.

## Effects of Phthalates on Folliculogenesis

### Effects of phthalates on oocyte development and primordial follicle assembly

Limited studies have investigated the effects of phthalates on folliculogenesis, but there is evidence suggesting that phthalates alter the formation and/or function of follicles at several stages of development. Specifically, phthalates have been shown to disrupt the earliest stages of folliculogenesis by altering ovarian and oocyte development. DEHP exposure in Japanese medaka during sexual development has been shown to inhibit oocyte development (112). When given in an aqueous solution at 1–50 μg/l from hatching to 3 months of age, DEHP exposure decreased the percentage of completely matured oocytes in the ovaries, most likely via an anti-estrogenic mechanism of action (112). MEHP exposure for 24 h at 250–500 μM has been shown to decrease murine fetal oocyte viability using an *in vitro* oocyte culture system (113). This decrease in oocyte survival is attributed to an alteration in oocyte oxidative stress as the mRNA levels of mitochondrial respiratory chain protein (*Nd1*) were decreased and the mRNA levels of Cu–Zn superoxide dismutase (*Sod1*) were increased in the oocytes following MEHP exposure (113). A decrease in *Nd1* mRNA may lead to an increase in reactive oxygen species (ROS), which are toxic to the oocyte and are also associated with an increased risk of infertility (114). The increase in the antioxidant *Sod1* mRNA is most likely a compensatory response in detoxifying the increased ROS following MEHP exposure. DEHP exposure further affects oocyte development by causing heritable modifications in DNA methylation in mouse oocytes (115). When given to pregnant mice during the length of gestation, DEHP exposure at 40 μg/kg/day reduced the methylation of CpG sites in the two critical imprinting genes, insulin-like growth factor 2 receptor (*Igf2r*) and paternally expressed gene 3 (*Peg3*), in the primordial germ cells of the fetal ovary at gestational day 12.5 and the oocytes of the offspring by post-natal day 21 (115). Interestingly, the decrease in oocyte DNA methylation of *Igf2r* and *Peg3* is also evident in the oocytes of the F2 offspring, suggesting that the effects of DEHP on oocyte development are heritable (115). Gestational exposure to a single intraperitoneal injection of DIBP resulted in architectural disarray of follicles in fetal rats (116). Specifically, DIBP exposure at 0.375–1.25 ml/kg increased the numbers of degenerated oocytes and empty follicles without oocytes, and the blood vessels located in the stroma of the ovary appeared prominent and congested (116). MEHP has also been shown to affect ovarian development in the human. Human ovaries from gestational weeks 7 to 12 were cultured with MEHP at 10^-4^ M for 72 h and had dysregulated lipid/cholesterol synthesis as evident by an increase in the mRNA levels of liver X receptor alpha (*LXRα*) and sterol regulatory element-binding protein (SREBP) members (117). Interestingly, oocyte numbers were not affected by MEHP treatment, but the same study suggests that phthalate toxicity to the developing human ovary may be mediated by nuclear receptor signaling (117). It appears that phthalates disrupt early ovarian and oocyte development potentially leading to oocyte death and abnormal ovarian architecture (112, 113, 116). The mechanisms by which phthalates alter the earliest stages of ovarian development appear to include an anti-estrogenic response (112), an increase in oxidative stress (113), and heritable modifications to the oocyte epigenome (115).

Phthalates have also been shown to affect germ cell nest breakdown and primordial follicle assembly. Newborn mouse ovaries cultured with DEHP for 72 h at 10–100 μM had an increase in oocytes contained in the germ cell nest, and there was a decrease in primordial follicle numbers (118). Thus, germ cell nest breakdown and primordial follicle assembly were inhibited following DEHP exposure. Additionally, DEHP exposure increased apoptosis in the oocytes indicated by an increase in TUNEL positive oocytes and increased mRNA levels of pro-apoptotic BCL-2-associated X protein (*Bax*) (118). Further, DEHP decreased the mRNA levels of other factors associated with oocyte survival and primordial follicle formation, such as LIM homeobox 8 (*Lhx8*), factor in the germline alpha (*Figla*), spermatogenesis and oogenesis helix-loop-helix (*Sohlh2*), and newborn ovary homeobox (*Nobox*) (118). Similar to previous reports, DEHP exposure affected oocyte DNA methylation by inhibiting the demethylation of CpG sites of *Lhx8*, a process required for early folliculogenesis (118). These effects on primordial follicle formation can have lasting effects on folliculogenesis and fertility because primordial follicles serve as the female’s reproductive potential (46).

### Effects of phthalates on follicles across development

Phthalates have also been shown to affect the rate in which primordial follicles are recruited to the growing population of follicles. In the adult mouse, oral exposure to DEHP for 10 and 30 days at 20 μg/kg/day–750 mg/kg/day accelerates primordial follicle recruitment, evident by a decrease in primordial follicles and an increase in primary follicles (119). The mechanism by which DEHP accelerates primordial follicle recruitment is likely via overactivation of the phosphatidylinositol 3-kinase (PI3K) signaling pathway, a pathway that regulates primordial follicle survival, quiescence, and recruitment. Specifically, DEHP exposure increased the ovarian mRNA levels of 3-phosphoinositide-dependent protein kinase-1 (*Pdpk1*), mammalian target of rapamycin complex 1 (*Mtorc1*), which are factors that drive primordial follicle recruitment, and decreased the mRNA levels of phosphatase and tensin homolog (*Pten*) and tuberous sclerosis 1 (*Tsc1*), which are factors that maintain primordial follicle quiescence (119). Additionally, DEHP exposure for 10 days increased phosphorylated protein kinase B (pAKT) protein in the whole ovary and in primordial and primary follicles, and decreased PTEN protein in the whole ovary, further suggesting that DEHP overactivates ovarian PI3K signaling to promote the acceleration of primordial follicle recruitment (119). Similar effects on primordial follicle recruitment were observed following DEHP exposure during early post-natal life in mice. Following hypodermic injections during early post-natal life, DEHP at 20–40 μg/kg/day accelerated folliculogenesis by decreasing primordial follicles and increasing preantral and antral follicles when the ovaries were observed on post-natal day 15 and 21 (120). Further, when the treated mice were allowed to breed, the F1 offspring had a similar decrease in primordial follicle numbers when the ovaries were observed in adulthood (120). MEHP exposure *in utero* also accelerates folliculogenesis in mice. Oral exposure to MEHP via gavage from gestational days 17–19 at 100–1000 mg/kg/day resulted in an increase in preantral and antral follicles in the F1 generation (121). These F1 females exposed to MEHP *in utero* also exhibited premature reproductive senescence by 1 month, likely attributed to the acceleration of folliculogenesis evident by the follicle count data (121). Phthalates appear to accelerate primordial follicle recruitment by decreasing primordial follicle numbers and increasing the numbers of more mature follicle types, and this effect is consistent across timing and duration of exposure and the doses of phthalates used. Because the primordial follicle reserve is non-renewable, the above effects on primordial follicle recruitment can impact a female’s reproductive lifespan.

In addition to the effects of phthalates on immature follicle types, phthalates have also been shown to target and adversely affect more mature follicles. Exposure to DEHP alone and in combination with benzo[a]pyrene (B[a]P) via oral gavage decreased the population of primary and secondary follicles, potentially via induced follicular atresia in adult rats (122). Specifically, DEHP alone (600 mg/kg/day) and in combination with B[a]P (10 mg/kg/day) induced granulosa cell apoptosis, resulting in an increase in the number of atretic follicles across developmental stages (122). Likewise, *in utero* and lactational exposure to DEHP from midgestation to weaning at 405 mg/kg/day increased the number of atretic preantral follicles in the rat offspring during adulthood (123). A similar effect of increased atresia in growing follicles was seen in adult marine medaka. DEHP in an aqueous solution at 0.1–0.5 mg/l increased the numbers of atretic late-stage follicles, resulting in reproductive dysfunction following exposure from hatching to adulthood (124). A reduction in the growing population of follicles was also observed in DEHP-exposed neonatal ovaries after transplantation into the kidney capsules of immunodeficient mice. Specifically, newborn mouse ovaries were cultured with DEHP for 72 h at 10–100 μM and were then transplanted into adult mice to observe if folliculogenesis was impaired. Contrary to control-treated transplanted ovaries, DEHP-treated transplanted ovaries had few, if any, growing follicles following 21 days post-transplantation (118). Further, preantral follicles from rats cultured with MEHP *in vitro* for 10 days at 10–80 μg/ml had a lower survival rate and decreased rate of development to the antral stage (125). Likewise, secondary follicles from rats cultured with MEHP at 100 μg/ml had suppression of follicular development accompanied by a decrease in follicular viability and an increase in granulosa cell apoptosis (126). Prior to development to the antral follicle stage, phthalates appear to target primary and preantral follicle to induce atresia at a wide range of doses. This effect on atresia is likely attributed to phthalate-induced apoptosis of granulosa cells (122, 126).

Phthalates also target mature antral follicles by adversely inhibiting their growth and maturation. Much of the work investigating the effects of phthalates on antral follicle growth utilizes the novel method of the whole antral follicle culture system (127, 128). Using this method, DBP exposure for 168 h at 1000 μg/ml has been shown to inhibit antral follicle growth (129). This inhibition of antral follicle growth is likely attributed to defects in the cell cycle, which is necessary for appropriate granulosa cell proliferation and follicle growth. Specifically, DBP exposure at 1–1000 μg/ml decreased mRNA levels of cyclin D2 (*Ccnd2*), cyclin E1 (*Ccne1*), cyclin A2 (*Ccna2*), and cyclin B1 (*Ccnb1*), and increased the mRNA levels of cyclin-dependent kinase inhibitor 1A (*Cdkn1a*) at a time-point prior to growth inhibition (129). DBP-treated follicles had greater numbers of cells in the G_1_ phase, fewer numbers of cells in the S phase, and a trend for fewer numbers of cells in the G_2_ phase, further indicating cell cycle arrest following 24 h of culture (129). These defects in antral follicle growth potentially lead to the observed increase in atresia in DBP-treated follicles (129). Both DEHP at 1–100 μg/ml and MEHP at 0.1–100 μg/ml also inhibit antral follicle growth *in vitro*. Specifically, both chemicals inhibit antral follicle growth following 72 h of culture, and this effect persists for the duration of the 96 h culture (130–132). Similar to DBP, DEHP at 100 μg/ml disrupts the cell cycle by decreasing the mRNA levels of *Ccnd2* and cyclin-dependent kinase 4 (*Cdk4*), and MEHP at 10–100 μg/ml disrupts the cell cycle by decreasing the mRNA levels of *Ccnd2*, *Ccne1*, and *Cdk4* (131, 132). Further, MEHP exposure at 1–100 μg/ml increased the mRNA levels of pro-apoptotic *Bax* and apoptosis-inducing factor, mitochondrion-associated, 1 (*Aifm1*) and decreased the mRNA levels of anti-apoptotic *Bcl2* and Bcl2-like 10 (*Bcl2l10*), leading to antral follicle atresia (131, 133). Interestingly, DEHP and MEHP likely inhibit antral follicle growth and induce atresia via a mechanism involving oxidative stress. Specifically, DEHP at 10 μg/ml and MEHP at 1–100 μg/ml increased ROS levels in the treated follicles (130, 131). This is accompanied by reduced expression and enzyme activity of SOD1 following DEHP exposure, and reduced expression and enzyme activities of SOD1 and glutathione peroxidase (GPX) following MEHP exposure (130, 131). Supplementing the DEHP- and MEHP-treated follicles with estradiol (1–10 nM) or the antioxidant *N*-acetyl cysteine (NAC; 0.25–1 mM) only partially protects the follicles from phthalate-induced growth inhibition (130–132), but estradiol supplementation rescues the antral follicles from MEHP-induced atresia (133). In additional studies, exposure to DEHP via oral gavage at 2 g/kg/day reduced preovulatory follicle size in rats, due to a reduced granulosa cell size and area (134). Phthalates appear to directly target the antral follicle to inhibit growth via cell cycle inhibition (129, 131, 132), induce of atresia (129, 131, 133), and increase oxidative stress (130, 131). Overall, the phthalate-induced inhibition of antral follicle growth can potentially impair ovulation and steroidogenesis (46, 101).

### Effects of phthalates on ovulation and the corpus luteum

The process of oocyte maturation during the peri-ovulatory period is also affected by phthalate exposure. DEHP exposure in an aqueous solution at 0.02–40 μg/l in zebrafish inhibited oocyte germinal vesicle breakdown, which is a process required for the resumption of meiosis prior to ovulation (135). This effect was accompanied by an increase in the levels of ovarian bone morphogenetic protein 15 (BMP15) and decreases in the levels of LHR and membrane progesterone receptors (mPRs), which are factors that drive oocyte maturation (135). Similar effects were seen using *in vitro* maturation assays with bovine oocytes. MEHP exposure at 5–100 μM to denuded oocytes and cumulus–oocyte complexes for 22–24 h reduced the number of oocytes that resumed meiosis, indicated by an increase in the number of oocytes still in the germinal vesicle stage, and reduced the number of oocytes that progressed to metaphase II (136, 137). Bovine oocytes exposed to MEHP at 50 μM during maturation also had a decrease in the mRNA levels of *CCNA2*, acid ceramidase 1 (*ASAH1*; an anti-apoptotic factor), and POU domain, class 5, transcription factor 1 (*POU5F1*; a factor responsible for pluripotency), which potentially led to the observed increased in apoptotic oocytes during culture (137). These defects in oocyte maturation resulted in increased instances of poor-quality early embryos (137). Similar to the bovine model, DEHP exposure at 0.12–1200 μM inhibited oocyte maturation in the horse following an *in vitro* maturation assay (138). Further, ROS levels and apoptosis were increased in the cumulus granulosa cells (138). Additionally, BBP exposure at 100 μM to FSH-matured (10 ng/ml) porcine cumulus–oocyte complexes inhibited cumulus cell expansion, a process required for normal ovulation, transport through the oviduct, and fertilization (139). In the mouse, MEHP exposure at 200–400 μM in a maturation assay increased the number of oocytes in the germinal vesicle stage and decreased the number of oocytes that resumed meiosis in metaphase II (140). A similar effect was seen with *in utero* and lactational exposure to DEHP in adult female offspring. Specifically, DEHP exposure through gestation and weaning at 0.05–5 mg/kg/day decreased the numbers of oocytes that reached meiosis II when the offspring were superovulated as adults (141). Further, mouse oocytes that were matured *in vitro* had increased metaphase II spindle abnormalities following *in vivo* exposure to DEHP at 20–40 μg/kg/day, indicating that phthalates have the potential to alter post-meiotic resumption maturation processes (120). Phthalates appear to inhibit germinal vesicle breakdown and resumption of meiosis in multiple different models, and these effects on oocyte maturation may be detrimental to ovulation and normal embryonic development.

Along with defects in oocyte maturation, phthalates disrupt the ovulatory process. Zebrafish exposed to DEHP in an aqueous solution at 0.02–40 μg/l had a significant reduction in ovulations likely attributed to a decrease in the mRNA levels of prostaglandin-endoperoxide synthase 2 (*Ptgs2*), which is an enzyme required for one of the final triggers of ovulation following the LH surge (135). Further, the injection of DEHP inhibits ovulation in rats following equine chorionic gonadotropin (eCG)-induced ovulation (15–30 IU). Specifically, DEHP exposure at 500 mg/kg/day decreased the total number of rats that ovulated in response to eCG treatment, and DEHP exposure reduced the total number of ovulated oocytes following eCG treatment (142). Likewise, oral exposure to DEHP via gavage during metestrus at 2 g/kg/day delayed or suppressed ovulation by the first proestrus/estrus in rats (134). In fact, 7 out of 10 rats did not ovulate by vaginal estrus in response to DEHP treatment (134). These studies suggest that DEHP exposure is capable of inhibiting ovulation by decreasing the transcription of LH surge-response genes, even when the ovulatory process is chemically induced.

Phthalates have also been shown to disrupt the luteal transition and/or target corpora lutea. Perinatal exposure to DINP at 20,000 ppm in the rat decreased the number of corpora lutea present in adulthood (143). Adolescent rats exposed to DEHP via oral gavage for 28 days at 150–500 mg/kg/day also had a decrease in corpora lutea numbers (144). A similar effect of decreased corpora lutea numbers is seen when DEHP alone (300–600 mg/kg/day) and in combination with B[a]P (10 mg/kg/day) is administered to adult rats (122). Because mechanistic studies were not conducted, it is unknown if the decreases in corpora lutea numbers are due to an inhibition of ovulation, an inhibition in the luteal transition, and/or a direct destruction of corpora lutea caused by phthalate exposure. However, previous studies have shown that phthalates inhibit ovulation (134, 135, 142).

A few studies suggest that phthalates may alter the functionality of corpora lutea. Marmosets exposed to DEHP via oral gavage from weaning to sexual maturity at 500–2500 mg/kg/day had abnormally large corpora lutea present in the ovary, which is a finding often seen in older female marmosets (145). This increase in corpora lutea size likely causes the observed increase in ovarian weights following DEHP exposure (145). Conversely, adult sheep treated with DEHP intramuscularly at 25–50 mg/kg/day had smaller corpora lutea and a decreased luteal phase of the estrous cycle (146). These data suggest that phthalates have the potential to disrupt post-ovulatory ovarian processes, but further work must be done to elucidate the differences observed in the two studies.

### Epidemiological links between phthalate exposure and alterations in folliculogenesis

Very few studies have investigated the link between phthalate exposure in humans and alterations in folliculogenesis. One study examined the association of phthalate exposure and prevalence of polycystic ovary syndrome (PCOS), which is a gynecological disorder often associated with infertility and the presence of large, cystic follicles incapable of ovulating. Interestingly, lower urinary levels of MEHP, MEP, MBP, and MBzP were associated with an increased likelihood of PCOS when compared to control patients and patients with higher levels of these phthalate metabolites (147). Similarly, in the Western Australian Pregnancy Cohort Study, maternal serum levels of MEP and the sum of all phthalate metabolites were negatively associated with PCOS in the daughters (148). However, this study did not investigate the prevalence of PCOS in the daughters whose mothers had low levels of phthalate metabolites. In the same study, maternal levels of MEP had a negative association with anti-Müllerian hormone (AMH), which is a hormone secreted by granulosa cells of maturing follicles to restrict primordial follicle activation (148). Based on the paucity of available information, further epidemiology studies are warranted in investigating the effects of phthalate exposure on folliculogenesis in the human.

**Figure 4:**
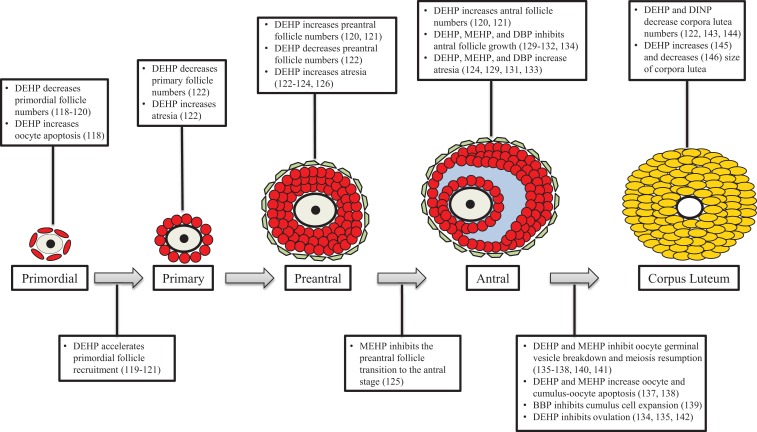
**Phthalates disrupt folliculogenesis**. This figure is a summation of the major findings on the effects of phthalates on folliculogenesis. Text boxes above a particular follicle type outline the major effects of phthalates at that stage of development, while text boxes below transition arrows outline the major effects of phthalates on that developmental transition.

Ovarian folliculogenesis is an essential process for normal reproductive and non-reproductive health, and increasing evidence suggests that phthalates have the ability to adversely affect this process in numerous aspects. Specifically, phthalates have been shown to disrupt ovarian/oocyte development, accelerate primordial follicle recruitment, target growing follicles, inhibit growth of antral follicles, disrupt oocyte maturation and ovulation, and alter post-ovulatory processes (Figure 4). The mechanisms by which phthalates exert these toxic effects on folliculogenesis are unknown, but a few studies have begun to elucidate these mechanisms and have shown that phthalates can modulate genes associated with folliculogenesis (Table 1). It is clear that the majority of work investigating the effects of phthalates on folliculogenesis focus solely on DEHP and its metabolite MEHP. Future work should elucidate the mechanisms by which DEHP and MEHP disrupt folliculogenesis and should incorporate exposures to other commonly used phthalates. Further, the doses used in the reviewed animal studies rarely encompass the range of estimated human exposure. It would be advantageous to conduct experiments with levels of phthalates that fall within the range of human exposure, especially considering that phthalates exhibit non-monotonic dose responses (149, 150). Additionally, experiments should be conducted to observe if these effects on folliculogenesis persist throughout the reproductive lifespan and if these effects directly cause infertility.

**Table 1:** **Genes associated with folliculogenesis that are altered by phthalate exposure**.

Phthalate (dose)	Model (duration of exposure)	Effect on gene (reference)	Gene name
DEHP (0.02–40 μg/l)	Adult zebrafish (21 days)	Decreased *Ptgs2* (135)	Prostaglandin-endoperoxide synthase 2
DEHP (100 μg/ml)	Mouse antral follicles (96 h)	Decreased *Ccnd2* (132)	Cyclin D2
		Decreased *Cdk4* (132)	Cyclin-dependent kinase 4
		Decreased *Sod1* (130)	Cu–Zn superoxide dismutase 1
DEHP (10–100 μM)	Neonatal mouse (72 h)	Increased *Bax* (118)	BCL-2-associated X protein
		Decreased *Lhx8* (118)	LIM homeobox 8
		Decreased *Figla* (118)	Factor in the germline alpha
		Decreased *Sohlh2* (118)	Spermatogenesis and oogenesis helix-loop-helix
		Decreased *Nobox* (118)	Newborn ovary homeobox
DEHP (20 μg/kg/day–750 mg/kg/day)	Adult mouse (10 or 30 days)	Increased *Pdpk1* (119)	3-phosphoinositide-dependent protein kinase-1
		Increased *Mtorc1* (119)	Mammalian target of rapamycin complex 1
		Decreased *Pten* (119)	Phosphatase and tensin homolog
		Decreased *Tsc1* (119)	Tuberous sclerosis 1
DEHP (40 μg/kg/day)	Fetal and prepubertal mouse, *in utero* (length of gestation)	Decreased methylation of *Igf2r* (115)	Insulin-like growth factor 2 receptor
		Decreased methylation of *Peg3* (115)	Paternally expressed gene 3
MEHP (1–100 μg/ml)	Mouse antral follicles (24–96 h)	Decreased *Ccnd2* (131)	Cyclin D2
		Decreased *Ccne1* (131)	Cyclin E1
		Decreased *Cdk4* (131)	Cyclin-dependent kinase 4
		Increased *Bax* (131)	BCL-2-associated X protein
		Increased *Aifm1* (133)	Apoptosis-inducing factor, mitochondrion-associated, 1
		Decreased *Bcl2* (131)	B-cell leukemia/lymphoma 2
		Decreased *Bcl2l10* (133)	Bcl2-like 10
		Decreased *Gpx* (131)	Glutathione peroxidase
		Decreased *Sod1* (131)	Cu–Zn superoxide dismutase 1
MEHP (10–4 M)	Human fetus (72 h)	Increased *LXRα* (117)	Liver X receptor alpha
		Increased *SREBP* members (117)	Sterol regulatory element-binding protein
MEHP (250–500 μM)	Fetal mouse oocytes (24 h)	Decreased *Nd1* (113)	Mitochondrial respiratory chain protein
		Increased *Sod1* (113)	Cu–Zn superoxide dismutase 1
MEHP (50 μM)	Bovine oocytes (22–24 h)	Decreased *CCNA2* (137)	Cyclin A2
		Decreased *ASAH1* (137)	Acid ceramidase 1
		Decreased *POU5F1* (137)	POU domain, class 5, transcription factor 1
DBP (1–1000 μg/ml)	Mouse antral follicles (24–168 h)	Decreased *Ccnd2* (129)	Cyclin D2
		Decreased *Ccne1* (129)	Cyclin E1
		Decreased *Ccna2* (129)	Cyclin A2
		Decreased *Ccnb1* (129)	Cyclin B1
		Increased *Cdkn1a* (129)	Cyclin-dependent kinase inhibitor 1A

## Effects of Phthalates on Steroidogenesis

### Effects of phthalate exposure on steroidogenesis *in vivo*

Gestational exposure to phthalates has been shown to alter steroidogenesis in female offspring. Oral exposure to MEHP via gavage from gestational days 17–19 at 100–1000 mg/kg/day increased the levels of serum FSH and estradiol in female mouse offspring once they reached adulthood (121). MEHP exposure *in utero* also decreased the mRNA levels of *Star* and *Cyp19a1* in the ovaries of the adult offspring (121). Estrous cyclicity, a process controlled by ovarian-derived hormones, was also altered in these offspring. MEHP-exposed females exhibited a delay in the onset of cyclicity, and MEHP exposure increased the time spent in estrus (121). Oral exposure to DIBP via gavage from gestational days 7–21 at 600 mg/kg/day increased anogenital distance in female rat offspring, a steroid hormone-regulated process, and increased the mRNA levels of *Cyp19a1* in the ovaries when the offspring were prepubertal (151). A similar effect of increased anogenital distance was observed in female offspring following gestational BBP exposure via oral gavage to rats at 500 mg/kg/day (152). Maternal exposure to DEHP in the diet at 0.05–5 mg/kg/day during the entire length of gestation through weaning decreased the mRNA levels of key steroidogenic enzymes and receptors in the ovaries of adult mouse offspring (141). Specifically, ovaries from the adult offspring had decreased levels of *Cyp19a1*, *Cyp17a1*, progesterone receptor (*Pgr*), FSH receptor (*Fshr*), and LHR (*Lhr*) (141). It is apparent that gestational exposure to phthalates results in defects in ovarian steroidogenesis across multiple developmental time-points by decreasing key steroidogenic enzyme levels.

Prepubertal exposure to phthalates has also been shown to disrupt ovarian steroidogenesis. DEHP exposure via inhalation from post-natal day 22–41 at 25 mg/m^3^ increased serum levels of cholesterol, LH, and estradiol in female rats following the duration of exposure (153). When the exposure window was expanded from post-natal day 22–84, DEHP exposure increased the mRNA levels of ovarian *Cyp19a1*, advanced the age of vaginal opening and first estrous cycle, and increased the number of irregular estrous cycles (153). The increase in *Cyp19a1* likely attributes to the increase in estradiol levels. Oral exposure to DEHP via gavage for 10 days at 500 mg/kg/day decreased the serum levels of progesterone and estradiol, and there was a trend of increased serum LH levels in prepubertal rats (154). Further, granulosa cells from DEHP-exposed prepubertal rats exhibited a decrease in *ex vivo* progesterone production even following FSH and LH stimulation, which was likely attributed to a decrease in the required transport of endogenous cholesterol into the mitochondria to initiate steroidogenesis (154). The discrepancy in the levels of steroid hormones following phthalate exposure is likely attributed to the route of exposure. When rats were exposed via inhalation, steroid hormone levels were increased (153). On the contrary, when rats were exposed via oral ingestion, steroid hormone levels were decreased (154). Further, the timing of exposure may explain why prepubertal-exposed animals had increased *Cyp19a1* levels (153), but *in utero*-exposed animals had decreased *Cyp19a1* levels (121, 141).

Additional *in vivo* studies indicate that phthalate exposure during adulthood targets the ovary and disrupts steroidogenesis. Exposure to DEHP via oral gavage for 8 days at 2 g/kg/day decreased serum estradiol levels in adult rats (134). This suppression of estradiol led to secondary rises in FSH levels and was unable to induce the LH surge needed for ovulation (134). Thus, DEHP exposure caused anovulation in the study (134). Further, DEHP exposure prolonged the duration of the estrous cycle in the adult rats (134). A similar study showed that DEHP exposure via oral gavage at 1000–3000 mg/kg/day also decreased serum estradiol levels in adult rats (155). In addition, serum testosterone, progesterone, LH, and FSH were also decreased following DEHP exposure (155). Similarly, DEHP exposure via oral gavage for 16 weeks at 500–2000 mg/kg/day prolonged the duration of estrous cycles, caused apoptosis and cell cycle arrest in granulosa cells, and decreased serum progesterone levels in adult mice (156). A similar effect on estrous cyclicity was seen where oral exposure to DEHP for 10 and 30 days at 20 μg/kg/day–750 mg/kg/day increased the amount of time adult mice spent in the estrous stage (119). Further, chronic DBP exposure from weaning, through puberty, mating, and gestation at 500–1000 mg/kg/day increased gestational *ex vivo* ovarian estradiol production and decreased gestational *ex vivo* ovarian progesterone production in adult rats (157). Conversely, DEHP exposure via intramuscular injections at 25–50 mg/kg/day increased plasma concentrations of progesterone in the adult ewe (146). DEHP exposure also decreased the duration of the ewe’s estrous cycle and increased the number of irregular estrous cycles (146). The effects on progesterone production and estrous cyclicity are likely attributed to DEHP toxicity on the corpora lutea (146). Estradiol- and progesterone-mediated processes, such as uterine decidualization, are also affected by exposure to phthalates in the adult rat. BBP, DBP, and MBP exposure via gastric intubation at 750–1500 mg/kg/day suppressed uterine decidualization in the adult rat, which is a required process for pregnancy and is controlled by ovarian-derived steroid hormones (158–160). Together, these studies provide evidence that phthalate exposure during adulthood alters ovarian steroidogenesis.

### Effects of phthalate exposure on steroidogenesis *in vitro*

Several *in vitro* studies using multiple culture models confirm and expand upon the ability of phthalates to disrupt ovarian steroidogenesis. Importantly, some of these studies also provide essential insight into the mechanisms by which phthalates disrupt steroidogenesis. Isolated ovarian cell cultures have shown that phthalates directly target specific cell types in the ovary and disrupt steroidogenesis in animal models. Specifically, MEHP exposure for 48 h at 50–200 μM suppressed estradiol production in rat granulosa cells (161–163). The decrease estradiol production from the granulosa cells was observed even with the supplementation of testosterone (a precursor for estradiol at 500 nM), FSH (an inducer and activator of aromatase for the conversion of testosterone to estradiol at 10 ng/ml), and 8-bromo cyclic adenosine monophosphate (a stable cAMP analog, which is a secondary messenger for FSH signaling in granulosa cells at 1 mM). Thus, MEHP exposure disrupts estradiol production independent of FSH–cAMP signaling (161). The mechanism by which MEHP suppresses estradiol in the culture system is via decreased mRNA levels, protein levels, and availability of aromatase (161, 162). Further, it is likely that MEHP acts through peroxisome proliferator-activated receptors (PPARs) to decrease aromatase transcription (163). PPARs are involved in granulosa cell differentiation, lipid metabolism, and even in the regulation of aromatase transcription and activity, and MEHP appears to activate PPARα and PPARγ in the granulosa cells to inhibit aromatase transcription (163). Further, MEHP exposure for 24 h at 100 μM decreased progesterone production and FSH-induced cAMP accumulation in rat granulosa cells (164). In contrast, MEHP exposure for 48 h at 100–250 μM in a different study increased basal steroidogenesis in rat granulosa cells, evident by increases in progesterone and protein levels of STAR (165). Perhaps the discrepancy in the MEHP-induced defects in steroidogenesis can be attributed to the dosage and use of different rat strains and the different susceptibilities to phthalate toxicity across strains. MEHP exposure inhibited steroidogenesis in Fisher 344 rat granulosa cells (161–164), but it stimulated steroidogenesis in Sprague-Dawley rat granulosa cells (165). This stimulation of steroidogenesis, evident by an increase in progesterone production, was also observed in KK-1 granulosa tumor cells exposed to MEHP for 24 h at 25–100 μM (166). Using another cellular model, DEHP exposure for 44 h at 1 μM increased the production of progesterone in FSH-matured porcine cumulus–oocyte complexes (139). This effect can potentially disrupt the final maturation processes of the oocyte following ovulation. In isolated bovine granulosa cells and isolated luteal cells, DEHP and MEHP exposure for 72 h at 0.1–10 ng/ml increased the production of oxytocin (167). Ovarian-derived oxytocin plays a role in the regulation of the estrous cycle. These studies show that phthalates have a direct effect on disrupting steroidogenesis in specific ovarian cell types in multiple animal models. The mechanism by which phthalates inhibit steroidogenesis in granulosa cells appears to be via suppression of PPAR-mediated aromatase transcription (161–163). The mechanism by which phthalates stimulate steroidogenesis in granulosa cells appears to be via increased steroidogenic enzyme levels (165). Future studies should aim to understand the differences in steroidogenesis in the different granulosa cell models.

Phthalates have also been shown to disrupt steroidogenesis in isolated human ovarian cell cultures. Similar to the previously mentioned study with rat granulosa cells (161–163), MEHP exposure at 0–500 μM/l decreased the production of estradiol in human granulosa-lutein cells isolated from women undergoing *in vitro* fertilization (168). Likewise, this inhibition of estradiol production is independent of FSH–cAMP signaling; thus, it is attributed to a decrease in the mRNA levels and activity of aromatase (168). In human luteal cells isolated from corpora lutea, DEHP, DBP, and BBP exposure for 24 h at 10^-6^–10^-9^ M decreased basal and human chorionic gonadotropin-stimulated progesterone production (169). In conjunction, DEHP, DBP, and BBP exposure decreased prostaglandin E2 (PGE2) secretion and DEHP decreased prostaglandin F2α (PGF2α) secretion from the luteal cells (169). Further, all three chemicals inhibited luteal cell release of vascular endothelial growth factor (VEGF) (169). Prostaglandins and VEGF are regulators of corpora lutea survival. Specifically, PGE2 and VEGF are luteotrophic factors and PGF2α is a luteolytic factor. Another study has shown phthalate-induced defects in immortalized human granulosa cell lines. In detail, BBP exposure at 1 μM in HO23 cells increased the mRNA and protein levels of aryl hydrocarbon receptor (AHR), aryl hydrocarbon receptor nuclear translocator (ARNT), and cytochrome-P450 1B1 (CYP1B1), which are involved in estradiol metabolism, resulting in reduced cell viability and potential decreases in estradiol, though this was not directly tested (170). Overall, phthalates appear to directly disrupt steroidogenesis by decreasing steroid hormone and steroidogenic enzyme levels in human ovarian cells in a manner similar to animal studies *in vitro*.

Expanding on the use of individual cell types, other culture systems utilizing the entire antral follicle and whole sections of ovaries have been used to investigate the effects of phthalates on steroidogenesis. This is important because steroidogenesis is a multi-cellular process involving both granulosa cells and theca cells. MEHP exposure for 48 h at 10–100 μg/ml increased the levels of progesterone and decreased the levels of androstenedione, testosterone, and estradiol in isolated rat secondary follicles (126). Interestingly, even with the decreases in the three sex steroid hormones, the increase in progesterone promoted an increase in the combined level of all steroid hormones in response to MEHP exposure (126). This suggests that MEHP potentially stimulates steroidogenesis in the secondary follicle, but it inhibits the conversion of progesterone to androstenedione (126). Further studies using mouse preantral follicles show that MEHP exposure at 10–200 μM increased the levels of progesterone, testosterone, and estrone (127). The discrepancies between testosterone production in these two studies can possibly be attributed to species differences and differences in culture methods. Using antral follicles, the most steroidogenically active follicle type, isolated from mice, DEHP (10–100 μg/ml) and MEHP (1–100 μg/ml) exposure for 96 h decreased estradiol production via inhibition of *Cyp19a1* transcription (132). This effect on steroidogenesis coincides with the DEHP- and MEHP-induced inhibition of antral follicle growth, cell cycle arrest evident by alterations in *Ccnd2*, *Ccne1*, and *Cdk4* mRNA levels, atresia evident by alterations in *Bax*, *Aifm1*, *Bcl2*, and *Bcl2l10* mRNA levels, and induction of oxidative stress evident by increases in ROS and altered SOD1 and GPX protein and activity (130–132). However, it is unknown if the inhibition of steroidogenesis causes these other toxic events or is a secondary response to defects in cell cycle progression and/or oxidative stress. Interestingly, supplementing the media with estradiol (1–10 nM) and NAC (0.25–1 mM), an antioxidant, only partially protected the antral follicle from DEHP- and MEHP-induced growth inhibition and *Cyp19a1* transcription (130–132), but estradiol completely rescued antral follicles from MEHP-induced atresia (133). This likely suggests that the effects of MEHP on estradiol production precede and promote the incidence of atresia (133). In another study, DBP exposure for 96 h at 1000 μg/ml decreased estradiol levels, and exposure for 168 h promoted atresia in cultured mouse antral follicles (129). Similar to DEHP and MEHP, this effect on steroidogenesis coincided with DBP-induced (1–1000 μg/ml) inhibition of antral follicle growth, cell cycle arrest evident by an increase in the number of follicular cells in the G_1_ stage and alterations in the mRNA levels of *Ccnd2*, *Ccne1*, *Ccna2*, *Ccnb1*, and *Cdkn1a*, and atresia evident by alterations in the mRNA levels of BH3 interacting-domain death agonist (*Bid*) and *Bcl2* (129). These studies suggest that the entire follicle unit is a target for phthalate-induced disruption of steroidogenesis. Studies using secondary and preantral follicles have shown an increase in steroid hormone levels following phthalate exposure (126, 127). Meanwhile, studies using more mature antral follicles have shown a decrease in steroid hormone levels following phthalate exposure, and the mechanisms by which phthalates inhibit steroidogenesis may involve an inhibition of antral follicle growth (129, 132), an induction of atresia (130–133), an increase in oxidative stress (130, 131), and decreases in steroidogenic enzyme levels (132).

Similar to the follicle culture, minced ovary cultures, containing all follicular cell types, have been used to investigate the effects of phthalates on steroidogenesis. DEHP exposure *in vivo* altered the steroidogenic profile of minced rat ovaries cultured for 1 h depending on the stage of the estrous cycle. Specifically, DEHP exposure at 1500 mg/kg/day increased the minced ovary production of testosterone and estradiol when the rats were euthanized in diestrus (171, 172). Conversely, when the rats were euthanized in estrus, cultured minced ovaries produced less estradiol (171, 172).

### Epidemiological links between phthalate exposure and alterations in steroidogenesis

Exposure to phthalates has been shown to disrupt ovarian steroidogenesis and steroidogenic-controlled processes. Though limited, there is epidemiologic evidence that phthalate exposure is associated with steroidogenic defects. Specifically in the Western Australian Pregnancy Cohort Study, serum from pregnant women during gestational week 18 was subjected to measurements of phthalate metabolites and hormones and the study found that several phthalate metabolites have a negative association with maternal sex hormone-binding globulin, and MEP had a negative association with AMH in the adolescent daughter (148). Further, the sum of DEHP metabolites was associated with a trend for an earlier age at menarche in the adolescent female offspring, which is a process heavily controlled by ovarian steroid production (148). In another study, urinary levels of MEHP and the oxidative monoester metabolite mono(2-ethyl-5-hydroxyhexyl) phthalate in mothers were negatively associated with free testosterone levels and the free testosterone to estradiol levels ratio in the cord serum from female human infants (173). Similarly, the urinary levels of several phthalate metabolites were associated with decreased serum total testosterone levels in women aged 6–20 and 40–60 years from the National Health and Nutrition Examination Survey (174). Further, urinary levels of DEHP metabolites and MBP were associated with decreased testosterone levels in pregnant women in the Study for Future Families (175). Conversely, *in utero* exposure to MEP and MBzP was associated with increased testosterone levels in girls at ages 8–13 years from the Mexico City birth cohort (176). Urinary levels of MEHP and MBzP in 8-year-old girls from Taiwan were also positively associated with increased serum progesterone levels and urinary levels of MBzP and MBP were positively associated with increased serum FSH levels (177).

**Figure 5:**
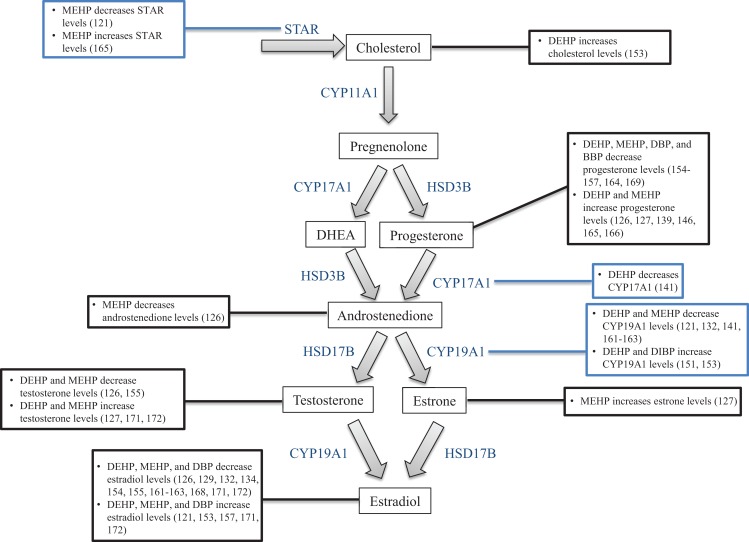
**Phthalates alter steroidogenesis**. This figure is a summation of the major findings on the effects of phthalates on steroidogenesis. Black text boxes connected to hormones outline the major effects of phthalates on the levels of that hormone. Blue text boxes connected to steroidogenic enzymes outline the major effects of phthalates on the mRNA and/or protein levels of that enzyme.

Proper regulation of ovarian steroidogenesis is vital for reproductive and non-reproductive health, and numerous studies indicate that phthalates have the ability to dysregulate steroidogenesis in multiple aspects (Figure 5). Specifically, phthalates have been shown to intervene in the production and secretion of multiple sex steroid hormones in both *in vivo* and *in vitro* systems to often lead to a decrease in estradiol levels. Further, phthalates have been shown to directly target several steroidogenic cell types in the ovary to elicit an adverse effect on steroid hormone production. These effects on steroidogenesis are likely attributed to alterations in the transcription of genes that synthesize and metabolize estradiol (Table 2). Importantly, the effects observed in animal studies moderately correlate to the effects observed in human ovarian cell types (161, 162, 164, 168, 169). As is the case with studies investigating the effects of phthalates on folliculogenesis, future work investigating the effects of phthalates on steroidogenesis should incorporate exposures to other commonly used phthalates to expand upon what is known regarding DEHP and MEHP, which are the two most extensively studied phthalates. Further, the doses used in these studies should fall within the range of estimated human exposure. Most often, the doses used in the reviewed studies exceed human exposure levels. Because phthalates exhibit a non-monotonic dose response, the effects of phthalates on steroidogenesis at lower levels may be more toxic and/or have different mechanisms than at higher levels (149, 150). Additionally, future work should elucidate the mechanisms by which phthalates disrupt steroidogenesis, and investigate whether the phthalate-induced disruption in steroidogenesis leads to infertility and non-reproductive complications.

**Table 2:** **Genes associated with steroidogenesis that are altered by phthalate exposure**.

Phthalate (dose)	Model (duration of exposure)	Effect on gene (reference)	Gene name
DEHP (0.05–5 mg/kg/day)	Adult mouse, *in utero* (length of gestation–weaning)	Decreased *Cyp19a1* (141)	Cytochrome-P450 aromatase
		Decreased *Cyp17a1* (141)	Cytochrome-P450 steroid 17-α-hydroxylase 1
		Decreased *Pgr* (141)	Progesterone receptor
		Decreased *Fshr* (141)	FSH receptor
		Decreased *Lhr* (141)	LH receptor
DEHP (100 μg/ml)	Mouse antral follicles (96 h)	Decreased *Cyp19a1* (132)	Cytochrome-P450 aromatase
DEHP (25 mg/m^3^)	Prepubertal rat (63 days)	Increased *Cyp19a1* (158)	Cytochrome-P450 aromatase
MEHP (10 μg/ml)	Mouse antral follicles (96 h)	Decreased *Cyp19a1* (132)	Cytochrome-P450 aromatase
MEHP (100–1000 mg/kg/day)	Adult mouse, *in utero* (gestational day 17–19)	Decreased *Star* (121)	Steroidogenic acute regulatory protein
		Decreased *Cyp19a1* (121)	Cytochrome-P450 aromatase
MEHP (50–200 μM)	Rat granulosa cells (48 h)	Decreased *Cyp19a1* (166–168)	Cytochrome-P450 aromatase
BBP (1 μM)	HO23 cells (24 h)	Increased *AHR* (175)	Aryl hydrocarbon receptor
		Increased *ARNT* (175)	Aryl hydrocarbon receptor nuclear translocator
		Increased *CYP1B1* (175)	Cytochrome-P450 1B1
DIBP (600 mg/kg/day)	Prepubertal rat, *in utero* (gestational day 7–21)	Increased *Cyp19a1* (156)	Cytochrome-P450 aromatase

## Summary and Future Directions

Phthalates are a group of EDCs that target the ovary to adversely affect the two essential processes of folliculogenesis and steroidogenesis. This is concerning for public health because phthalates are used extensively in a wide variety of commonly used items, resulting in ubiquitous human exposure. Phthalates have been shown to alter ovarian and oocyte development, target specific follicle types, alter progression of follicular development, and disrupt the functionality of follicles and corpora lutea. Specifically, phthalates have been shown to inhibit germ cell nest breakdown and primordial follicle assembly (118), accelerate primordial follicle recruitment (119–121), inhibit antral follicle growth (129–132) and final oocyte maturation (120, 135–141) to potentially inhibit ovulation (134, 142), and induce atresia in follicles across several stages of development (122–124, 129–131, 133). Further, increasing evidence suggests that phthalates disrupt the production, secretion, and action of several essential sex steroid hormones via altered mRNA, protein, and activity of multiple steroidogenic enzymes. These effects most commonly result in decreased estradiol levels (126, 129, 132, 134, 154, 155, 161–163, 168, 171, 172); however, some studies suggest that phthalates stimulate steroidogenesis (121, 126, 139, 146, 165, 171, 172). Regardless, these effects on folliculogenesis and steroidogenesis can have lasting effects on reproductive and non-reproductive health, as both of these processes are essential for fertility, maintenance of appropriately timed reproductive senescence, and the regulation of skeletal, cardiovascular, and brain health.

Further study is warranted in investigating the effects of phthalates on ovarian function. In particular, researchers should expand upon the dose ranges used in their studies to incorporate doses that mimic human exposure. The majority of the cited work focuses primarily on doses that exceed the range of estimated human exposure. Although these findings are important, observing the effects at levels that mimic human exposure would increase the translational nature of studies. Further, a unique characteristic of EDCs is that low doses often elicit different or more profound effects than high doses (149, 150). To expand upon the use of doses that mimic human exposure, future studies should also consider realistic routes and lengths of exposure. Humans are predominantly exposed to phthalates via oral ingestion, not gavage, and are exposed throughout the duration of the day, not a single bolus. Thus, studies should utilize oral dosing or exposure in the diet over multiple time-points in the day. Humans are also chronically exposed to phthalates from gestation through adulthood. Therefore, studies should investigate the effects of phthalate exposure, starting *in utero* and continuing in adulthood, on ovarian function across all stages of development. Additionally, humans are exposed to multiple phthalates and other environmental toxicants daily. The majority of the cited work understandably focuses on single phthalate exposures, but future studies should incorporate exposures to phthalate mixtures as well as a mixture of phthalates and other ubiquitous toxicants. The above suggestions would aid in translating the findings from animal studies to potential effects in humans. These future studies should also focus on the mechanisms of phthalate-induced ovotoxicity. Some studies suggest that phthalates exert toxicity via an estrogenic, anti-estrogenic, oxidative stress response, or PPAR activation depending on the model system, dose, and exposure window. However, further work must be done to elucidate the mechanisms by which phthalates disrupt folliculogenesis and steroidogenesis. This will aid in the treatment and/or prevention of phthalate-induced reproductive diseases.

## Author Contributions

Both Patrick R. Hannon and Jodi A. Flaws contributed to the concept, analysis, and interpretation of the structure and data within this review. Patrick R. Hannon and Jodi A. Flaws critically drafted and revised the review for intellectual content. Both Patrick R. Hannon and Jodi A. Flaws have approved of the final version to be published. Patrick R. Hannon and Jodi A. Flaws agree to be accountable for all aspects of the review.

## Conflict of Interest Statement

The authors declare that the research was conducted in the absence of any commercial or financial relationships that could be construed as a potential conflict of interest.
